# Human papillomavirus type 16 E6 variants in France and risk of viral persistence

**DOI:** 10.1186/1750-9378-8-4

**Published:** 2013-01-23

**Authors:** Iris Cornet, Tarik Gheit, Gary M Clifford, Jean-Damien Combes, Véronique Dalstein, Silvia Franceschi, Massimo Tommasino, Christine Clavel

**Affiliations:** 1 International Agency for Research on Cancer, 150 cours Albert Thomas, Lyon, 69372 Cedex 08, France; 2 INSERM UMR-S 903 / Université de Reims Champagne-Ardenne / CHU Reims, Laboratoire Pol-Bouin, Reims, France

**Keywords:** Cervical cancer screening, Cohort, Polymorphism, HPV, Variants, Persistence

## Abstract

**Background:**

Only a small portion of HPV 16 infections persist and can lead to cervical intraepithelial lesions and cancer. Factors that favour HPV persistence versus clearance are still poorly understood, but several studies have suggested that HPV intra-type variants may influence persistence and clinical outcome. The aim of this study was to assess the possible association between HPV 16 variants and the risk for viral persistence in the general population of France.

**Methods:**

One hundred and forty two women infected with HPV 16 with normal cytology, without previous treatment for cervical lesions, and with a valid second follow-up visit 4 to 16 months later, were selected from patients participating in routine cervical cancer screening in the Reims HPV Primary Screening Cohort Study. HPV intra-type variants were determined by sequencing the HPV 16 E6 open reading frame, and were compared for viral persistence at the second visit using odds ratios (OR) to estimate relative risk.

**Results:**

Although no statistically significant differences in risk for persistence were observed by the HPV 16 variant lineage, European variants containing the polymorphism 350 T (EUR-350 T) appeared to persist more often than those containing 350 G (EUR-350 G) (OR = 1.6, 95% CI = 0.8-3.4).

**Conclusions:**

No strong differences were observed in the risk of viral persistence for the HPV 16 variants that predominate in France.

## Background

High-risk (HR) human papillomaviruses (HPVs), are the etiological agents of cervical cancer [1]. Even though infection with an HR-HPV is frequent in sexually active women, the majority of infections are resolved by the immune system in 6–12 months [2]. Only a small fraction of infections persist and can lead to cervical intraepithelial neoplasia (CIN), which may progress to cervical cancer. Factors that favour HPV persistence versus clearance, other than HPV type itself, are still poorly understood, but several studies have suggested that HPV intra-type variants could influence persistence and clinical outcome [3-8].

A recent proposal to define major HPV variant lineages is by approximately 1.0% difference between full genomes of the same HPV type, with differences of 0.5-0.9% designated as sub-lineages [9-11]. Naturally occurring genetic variants of HPV 16 are common and have previously been classified into 4 major lineages; European-Asian, including the sub-lineages European (EUR), and Asian (As), African 1 (AFR1), African 2 (AFR2) and North-American/Asian-American (NA/AA). The names of the (sub)lineages derive from the geographical origin of the populations in which they were originally isolated [12].

The most common individual polymorphism within the European lineage is T350G in the E6 oncogene, which leads to an amino acid change Leucine to Valine (L83V). Thus, the European lineage can be divided into isolates containing 350 T (EUR-350 T) or 350 G (EUR-350 G), even though this polymorphic position does not seem to define phylogenetic lineages [10]. Several studies suggest that the EUR-350 T and EUR-350 G have a different distribution among cervical cancer cases and controls and can influence persistence [3,13,14].

The aim of this study was to assess the possible association between the genetic variants of HPV 16 and the risk of persistent HPV infection in the general population in France.

## Results

Sequencing results were obtained for a total of 142 women infected with HPV 16 with normal cytology and a valid follow-up visit. The follow-up visits ranged from minimum 4.0 months to maximum 16.0 months (median: 7.7 months, 25th percentile: 6.2 months, 75th percentile: 10.9 months). The majority of the baseline infections belonged to the European lineage (n = 131, 92.3%), with EUR-350 T and EUR-350 G accounting for 40.8% and 51.4% of the isolates, respectively. C109T (n = 3) was the only other single nucleotide polymorphism (SNP) found in more than one EUR isolate (Table 1). The number of non-EUR isolates was small (n = 11) and consisted of African-lineages and NA-lineage isolates (Table 1).

**Table 1:** Nucleotide sequence variations in the HPV 16 E6 gene of 142 HPV 16-positive French women

**Status**		**HPV 16 E6 nucleotide position**																								
Clearance*	Persistence*	**109**	**131**	**132**	**143**	**145**	**176**	**183**	**187**	**196**	**255**	**256**	**269**	**275**	**285**	**286**	**289**	**295**	**335**	**350**	**403**	**453**	**464**	**478**	**532**	**(sub-) lineage**
**N**	**N**	**T**	**A**	**G**	**C**	**G**	**G**	**T**	**A**	**G**	**G**	**C**	**G**	**A**	**C**	**T**	**A**	**T**	**C**	**T**	**A**	**G**	**A**	**C**	**A**	**Reference**
13	29	-	-	-	-	-	-	-	-	-	-	-	-	-	-	-	-	-	-	-	-	-	-	-	-	EUR-350 T
6	6	0	0	0	0	0	0	0	0	0	0	-	-	-	-	-	-	-	-	-	0	0	0	0	0	EUR-350 T
0	1	-	-	-	-	-	C	-	-	-	-	-	-	-	-	-	-	-	-	-	-	-	-	-	-	EUR-350 T
0	1	-	-	-	-	-	-	-	G	-	-	-	-	-	-	-	-	-	-	-	-	-	-	-	-	EUR-350 T
0	1	-	-	-	-	-	-	-	-	-	-	-	A	-	-	-	-	-	-	-	-	-	-	-	-	EUR-350 T
0	1	-	-	-	-	-	-	-	-	-	-	-	-	-	-	-	-	-	-	-	-	-	G	-	-	EUR-350 T
22	27	-	-	-	-	-	-	-	-	-	-	-	-	-	-	-	-	-	-	G	-	-	-	-	-	EUR-350 G
9	9	0	0	0	0	0	0	0	0	0	0	-	-	-	-	-	-	-	-	G	0	0	0	0	0	EUR-350 G
0	3	C	-	-	-	-	-	-	-	-	-	-	-	-	-	-	-	-	-	G	-	-	-	-	-	EUR-350 G
0	1	-	-	-	-	-	-	-	-	-	-	-	-	-	-	-	-	-	-	G	-	T	-	-	-	EUR-350 G
0	1	-	-	-	-	-	-	-	-	-	-	-	-	-	-	-	-	-	-	G	-	-	-	A	-	EUR-350 G
1	0	-	-	-	-	-	-	-	-	-	-	-	-	G	-	-	-	-	-	G	-	-	-	-	-	EUR-350 G
0	1	-	-	-	-	T	-	-	-	-	-	-	-	-	-	A	G	-	T	G	-	-	-	-	-	NA (non-EUR)
1	1	-	-	C	G	T	-	-	-	-	-	-	-	-	-	A	G	-	T	-	-	-	-	-	-	AFR1 (non-EUR)
0	1	-	G	-	G	T	-	-	-	-	-	-	-	-	-	A	G	G	T	G	-	-	-	-	-	AFR1 (non-EUR)
2	1	C	-	T	G	T	-	-	-	-	-	-	-	-	-	A	G	-	T	-	G	-	-	-	-	AFR2 (non-EUR)
1	0	C	-	T	G	T	-	C	-	-	-	-	-	-	-	A	G	-	T	-	G	-	-	-	-	AFR2 (non-EUR)
0	1	C	-	T	G	T	-	-	-	-	-	-	-	-	G	A	G	-	T	-	G	-	-	-	-	AFR2 (non-EUR)
0	1	-	-	-	G	T	-	-	-	A	T	T	-	-	-	A	G	G	T	-	-	-	-	-	-	AFR2 (non-EUR)
1	0	-	-	-	G	T	-	-	-	-	-	-	-	-	-	A	G	-	T	-	-	-	-	-	-	AFR2 (non-EUR)
56	86																									

Sequence results are shown by outcome of infection: *Clearance (HPV16-negative at follow-up visit between 4–16 months) or *Persistence (HPV16-positive at follow-up visit between 4–16 months).

Abbreviations: *EUR* EURopean, *NA* North American, *AFR* AFRican.

0 = failed sequencing for the whole E6 gene, a shorter fragment of E6 was determined (nt. 256–400).

Of these 142 women infected with HPV 16, 86 (60.6%) showed a persistent infection at the second visit. The proportion of HPV 16 infections that persisted was slightly higher for EUR-350 T (67.2%) than EUR-350 G (56.1%) (OR = 1.6, 95% CI = 0.7-3.5), although this difference did not reach statistical significance. Adjustment for age and time to second visit did not materially alter this finding (OR = 1.6, 95% CI = 0.8-3.4) (Table 2). Among the 32 HPV 16 infections that had evidence of persistence for 12 months and more, 16 (50.0%) were EUR-350 T and 13 (40.6%) EUR-350 G (OR versus clearance =2.1, 95% CI = 0.7-5.8, data not shown).

**Table 2:** Outcome of HPV 16 infection in 142 French women, by HPV 16 variant lineage

**Variant lineage**	Clearance*	Persistence*	
	**n (%)**	**n (%)**	**aOR**^**†**^**[95% CI]**
EUR-350 G	32 (43.8)	41 (56.1)	1
EUR-350 T	19 (32.8)	39 (67.2)	1.6 [0.8 – 3.4]
Non-EUR	5 (45.5)	6 (54.5)	0.9 [0.2 – 3.4]

* Clearance = HPV 16-negative at follow-up visit; Persistence = HPV 16-positive at follow-up visit.

^†^ aOR: Odds Ratio adjusted for age and time interval between first and follow-up visit.

Due to the rarity of non-European lineages a comparison by individual lineage could not be made. However, the proportion of all non-European lineages combined that persisted was similar to that for EUR-350 G (OR = 0.9, 95% CI = 0.2-3.4) (Table 2).

## Discussion

There were no statistically significant differences in the risk for persistence by HPV 16 variant lineage among women participating to the Reims HPV Primary Screening Cohort Study between 2001 and 2008. This finding, together with the distribution of the HPV 16 variants among infections at baseline, is consistent with results of a smaller and earlier study from this same cohort [14]. Nevertheless, European lineage isolates containing the polymorphism 350 T (EUR-350 T) did appear to persist more often than those containing 350 G (EUR-350 G) (OR = 1.6, 95% CI = 0.8-3.4). This is consistent with the findings of the only other population-based study of HPV 16 variant persistence in Europe: among 261 HPV 16 infections in a large cohort in Denmark, infection with EUR-350 T was associated with a significant increased risk for persistence at 2 years (OR versus EUR-350 G = 2.06, 95% CI = 1.04 - 4.25) [3].

One weakness of the present study was the inability to make any robust reflection on viral persistence of non-EUR HPV 16 lineages, due to their rarity in the French population [4,7,15]. Furthermore, the time-interval to determine infection persistence was relatively short, driven by the use of samples from a real-life screening practice in which women with an HR-HPV infection with normal cytology were advised to return at 6–12 months.

As a consequence of viral persistence, it would be expected to find the EUR-350 T more often in cervical cancer. In accordance, Zehbe et al. showed an enrichment of EUR-350 T in invasive cervical cancer in the Czech Republic, and, to a lesser extent, in Italy. However, they also found a significant under-representation of EUR-350 T in invasive cervical cancer in Sweden [13]. This suggests that the relative oncogenic potential of HPV 16 EUR-350 T versus EUR-350 G might be population-specific [13].

## Conclusions

Both EUR-350 T and EUR-350 G are found commonly in precursor lesions and cervical cancers in Northern Europe, meaning that any clinical utility of variant analysis is not yet evident. Nevertheless, understanding the genetic basis of differences in persistence of HPV 16 variants may help to unravel important interactions between HPV 16 and the host immune system. This could lead to better tools to control HPV infections and its associated malignancies.

## Methods

### Study population

Samples were selected from patients participating in the Reims HPV Primary Screening Cohort Study conducted in clinics and hospitals in Reims, France, and its surroundings [16,17]. Briefly, since 1997, women participating in the study were offered systematic cervical cancer screening combining liquid based cytology and HR-HPV testing by the Hybrid Capture 2 Test (Qiagen, Gaithersburg, MD USA) when they had their routine cervical smear performed. Women positive for HR-HPV or with abnormal cytology were advised to return 6–12 months later. For research purposes, since 2001, DNA has been extracted routinely from HR-HPV positive samples and screened for HPV 16 presence. The Reims HPV Primary Screening Cohort Study has been approved by the French committee “Comité de Protection des Personnes” (CPP-Est III, Nancy), statement no DC-2008-374. All women were informed and gave their written consent to participate in the study.

For the period 2001 to 2008, HPV 16-positive women that met the following criteria were selected: diagnosed with normal cytology and with a valid stored DNA sample at their first HPV 16-positive visit, without previous treatment for cervical lesions, and with a valid follow-up visit of known HPV 16 DNA between 4 to 16 months later.

These patients overlap partially (n = 21) with those included in a previous report on HPV 16 variants from this same cohort between 1997 and 2003 [14].

### HPV testing and sequencing

HPV 16 E6 variants were analysed from DNA stored from the first HPV 16-positive normal cytology sample. DNA was extracted from liquid-based cervical samples, collected in PreservCyt Medium (Hologic, Marlborough, MA USA), with the EZ1 DNA Tissue Kit on the EZ1 instrument (Qiagen, Hilden, Germany), according to the manufacturer’s instructions. Samples were lysed overnight using proteinase K at 56°C and DNA was eluted in a 200 μL volume.

HPV 16 DNA presence was screened initially by a specific HPV 16 E6/E7 Taqman real-time polymerase chain reaction (PCR) and confirmed by either the Linear Array HPV Genotyping Test (Roche Molecular Diagnostics, Branchburg, NJ USA) or the Inno-Lipa Extra HPV Genotyping Test (Innogenetics, Gent, Belgium).

The PCR and sequencing of the HPV 16 E6 gene was performed as described by Gheit et al. 2011 [3]. If amplification of the whole HPV 16 E6 failed, a shorter fragment of E6 (nt. 256 – 400) was amplified with the primers: 5’- GACTTTGCTTTTCGGGATT -3’ and 5’- GGCTTTTGACAGTTAATAC -3’. The sequences were analyzed using the Blast function from PubMed and single nucleotide polymorphisms (SNP) were identified using the prototype HPV 16 sequence described by Myers et al. [18]. Novel SNPs were confirmed by a second, independent PCR and sequencing reaction. Data were validated by comparing the SNPs reported by visual inspection in Blast with the output files from the DNA sequencer, using an ad hoc designed computer program.

### Statistical analysis

The HPV 16 infection was defined as persistent if the woman was still HPV 16-positive at the next follow-up visit. Relative risks for persistence were compared between EUR-350 T, EUR-350 G, and non-European lineages, using odds ratios (OR) and corresponding 95% confidence intervals (CI). ORs were adjusted for age (<28 and 28+ years) and the duration between two visits (<8 and 8+ months).

## Abbreviations

HR-HPV: High Risk Human Papillomavirus; CIN: Cervical Intra-epithelial Neoplasia; EUR: European; As: Asian; AFR: African; NA/AA: North-American / Asian-American; PCR: Polymerase Chain Reaction; SNP: Single Nucleotide Polymorphism; OR: Odds Ratio; CI: Confidence Interval.

## Competing interests

The authors declare that they have no competing interests.

## Authors’ contributions

VD and CC are responsible for the Reims screening study and research databases and participated in the study design. IC performed the HPV variants testing and lead drafting of the manuscript. TG developed the HPV variant testing platform and performed variant testing. MT developed the HPV variant testing platform and participated in the data interpretation. JC participated in the data analysis and performed statistical analysis. GC participated in the study design and interpretation. SF participated in study interpretation. All authors read and approved the final manuscript.
